# Evaluation of critical design parameters for RT‐qPCR‐based analysis of multiple dUTPase isoform genes in mice

**DOI:** 10.1002/2211-5463.12654

**Published:** 2019-05-29

**Authors:** Gergely A. Rácz, Nikolett Nagy, Zoltán Gál, Tímea Pintér, László Hiripi, Beáta G. Vértessy

**Affiliations:** ^1^ Institute of Enzymology, RCNS Hungarian Academy of Sciences Budapest Hungary; ^2^ Department of Applied Biotechnology and Food Sciences Budapest University of Technology and Economics Budapest Hungary; ^3^ Department of Animal Biotechnology, Agricultural Biotechnology Institute National Agricultural Research and Innovation Centre Gödöllő Hungary; ^4^ Faculty of Agricultural and Environmental Science Szent István University Gödöllő Hungary

**Keywords:** dUTPase, isoform‐specific expression levels, mitochondrial dUTPase isoform, mouse organs, nuclear dUTPase isoform, RT‐qPCR optimization

## Abstract

The coupling of nucleotide biosynthesis and genome integrity plays an important role in ensuring faithful maintenance and transmission of genetic information. The enzyme dUTPase is a prime example of such coupling, as it generates dUMP for thymidylate biosynthesis and removes dUTP for synthesis of uracil‐free DNA. Despite its significant role, the expression patterns of dUTPase isoforms in animals have not yet been described. Here, we developed a detailed optimization procedure for RT‐qPCR‐based isoform‐specific analysis of dUTPase expression levels in various organs of adult mice. Primer design, optimal annealing temperature, and primer concentrations were specified for both nuclear and mitochondrial dUTPase isoforms, as well as two commonly used reference genes, GAPDH and PPIA. The linear range of the RNA concentration for the reverse transcription reaction was determined. The PCR efficiencies were calculated using serial dilutions of cDNA. Our data indicate that organs involved in lymphocyte production, as well as reproductive organs, are characterized by high levels of expression of the nuclear dUTPase isoform. On the other hand, we observed that expression of the mitochondrial dUTPase isoform is considerably increased in heart, kidney, and ovary. Despite the differences in expression levels among the various organs, we also found that the mitochondrial dUTPase isoform shows a much more uniform expression pattern as compared to the reference genes GAPDH and PPIA.

**Abbreviations**
bp: base pair
cDNA: complementary DNA
C_q_: quantification cycle
dUMP: deoxyuridine 5'‐monophosphate
dUTP: deoxyuridine 5'‐triphosphate
dUTPase: deoxyuridine 5'‐triphosphate pyrophosphatase
GAPDH: glyceraldehyde 3‐phosphate dehydrogenase
PCR: polymerase chain reaction
PPIA: peptidyl‐prolyl cis‐trans isomerase A
qPCR: quantitative real‐time PCR; RT, reverse transcription

The dUTPase enzyme family is essential in maintaining genome integrity 1. It has been also found to be almost ubiquitous among free‐living organisms from bacteria to man and is encoded in diverse evolutionary strains of herpes viruses and retroviruses as well 2. The function of the enzyme is to remove dUTP from the cellular pool via its enzymatic action to catalyze pyrophosphorolysis of dUTP to result in dUMP and pyrophosphate products. This reaction has dual importance for cellular physiology, since on the one hand it sanitizes the nucleotide pool, while on the other hand it also produces dUMP, the key metabolite for *de novo* thymidylate biosynthesis. The significance of low dUTP level within the cells can be appreciated in light of the cellular response to increased concentration of uracil in DNA. Uracil can appear in DNA by two routes, either via cytosine deamination (that may occur spontaneously or through regulated cytosine deaminases) or thymine‐replacing incorporation under high cellular dUTP level. Cytosine deamination is a mutagenic reaction that will lead to an exchange of a G:U base pair to an A:T base pair if not promptly repaired. Thymine‐replacing uracils are not mutagenic *per se*; however they may perturb those DNA–protein interactions where the methyl group on the thymine base plays an important role. Both uracils from deaminated cytosines or thymine‐replacing incorporation are targeted by uracil excision repair (base excision repair, BER). If dUTP levels are high in the cellular milieu, repair synthesis will reintroduce uracils, creating a hyperactive futile cycle leading to chromosome fragmentation and cell death (thymine‐less cell death). Correct and efficient action of dUTPase is therefore of crucial importance to prevent thymine‐less cell death 3, 4.

It has been well established that dUTPase action is of most significance in cells with actively ongoing DNA synthesis. In eukaryotic organisms, dUTPase is usually present in two isoforms, one of these is nuclear and the other is mitochondrial/cytoplasmic 5, 6, 7, 8. In agreement with its physiological role, in *in vitro* cellular experiments the expression of the nuclear isoform of dUTPase was shown to be under cell cycle control. However, it is also known that in human cells the mitochondrial isoform is constitutively expressed. Although the expression pattern of dUTPase and its regulation in isolated eukaryotic cell lines has been covered in insightful details in numerous articles, much less is known about the expression pattern of dUTPase in living organisms. There is especially a lack of information regarding the expression pattern of the two distinct isoforms, since expression databases provide only a combined value. In plants, dUTPase level was associated with the mitotic status of meristem tissues 9. In Drosophila melanogaster, the expression of dUTPase was shown to be under developmental control 5, 10. In cancer tissues, overexpression of the nuclear isoform of dUTPase was associated with poor prognosis 11, 12, 13.

While there is a lack of detailed focused investigations, the Expression Atlas database obtained by large‐scale systems biology approaches provide a wealth of data on dUTPase expression levels 14, 15, 16, 17, 18. The reliability of such data is limited, and conflicting results can be found. Moreover, none of the data provide any specific information for both of the isoforms of dUTPases.

In light of the physiological importance of dUTPase and lack of data on its isoform‐specific expression level, our aim was to develop a highly reliable and well‐controlled reverse transcription/quantitative real‐time polymerase chain reaction (RT‐qPCR) method that can be used for parallel determination of the expression levels of both dUTPase isoforms. We selected mouse as an animal model for these studies. In mice, the two isoforms are generated via alternative spicing, thus for isoform‐specific detection, amplification of the differing regions of the two transcripts was necessary. Both of these regions have high GC content and therefore a complex secondary structure that makes the optimization process rather difficult 19. Despite the significance of RT‐qPCR, there has been a lack of consensus on how to perform and interpret experiments using this method. This issue was addressed by the MIQE guidelines in order to provide more reliable results 20. Here, we present a detailed optimization procedure for the RT‐qPCR conditions and report the performance parameters of the optimized method in compliance with the MIQE guideline. We also use this protocol to characterize the organ‐specific expression patterns of dUTPase isoforms in adult mice.

## Results and Discussion

### Optimization of RT‐qPCR conditions

#### Primer selection and annealing temperature optimization

**Figure 1:**
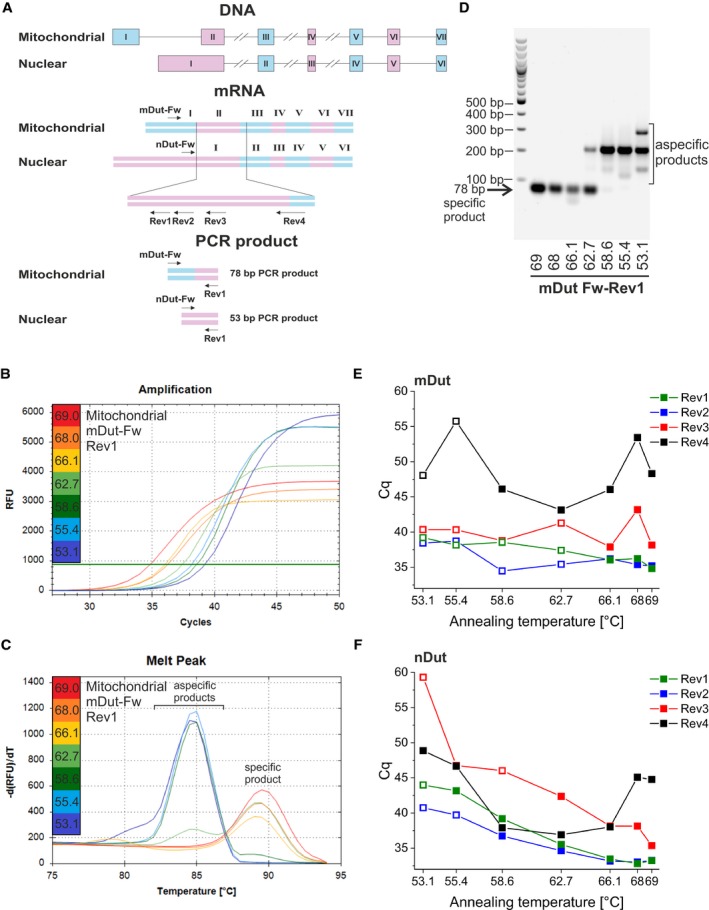
Selection of primers. (A) On the top, genomic sequences of both nuclear and mitochondrial isoforms are shown. Exons are indicated with Roman numerals in rectangles and shown with alternating blue and pink colors; introns are simplified as lines (for longer introns, lines are broken). In the middle, schematic illustration of primer candidates for amplification of both isoforms—after reverse transcription—of the *Dut* transcript is depicted. Exons are numbered and shown with alternating blue and pink colors. Common part of the sequence where the reverse primers are located is enlarged. All primers are indicated with arrows. On the bottom, PCR products amplified with the Rev1 reverse and the appropriate forward primers for both isoforms are shown. (B) Amplification curves for the mitochondrial isoform with the Rev1 reverse primer performed at a range of annealing temperatures from 53.1 to 69 °C using a gradient thermal block. RFU, relative fluorescence unit. (C) Melting curve analysis of the PCR products of the amplification introduced in panel B. (D) Agarose gel electrophoresis of the PCR products of the amplification introduced in panel B. The specific product is indicated with an arrow. (E) Quantification cycles (C_q_) at a range of annealing temperature from 53.1 to 69 °C with all four reverse primer candidates for the mitochondrial isoform. The solid squares indicate specific products as determined with agarose gel electrophoresis and melting curve analysis. Open squares indicate aspecific products. (F) The same representation for the nuclear isoform as shown in panel E

The *Dut* gene encodes for both nuclear and mitochondrial isoform of the enzyme dUTPase in mouse. These isoforms differ only in the first exon via alternative splicing (Fig. 1A). For the quantification of these transcripts, forward primers were designed in the first different exons. As opposed to the limited possibility for the forward primer design, several reverse primers could be applicable. Since the amplicon length can remarkably influence the efficiency of PCR, with short segments characterized with more efficient amplification, we deserved to amplify short regions of cDNA 21. The design of primers and the prediction of the specificity were carried out with the Primer‐BLAST tool 22. Unfortunately, *in silico* prediction of the specificity of the products with each potential primer pairs did not result in a conclusive optimal selection of adequate primers in terms of specificity. Therefore, we decided to select three exonic (Rev1, Rev2, and Rev3) and one exon junction spanning (Rev4) common reverse primers for both isoforms to be experimentally investigated (Table 1).

**Table 1:** Sequences and melting temperatures of the primers used in this study. n*Dut*‐Fw and m*Dut*‐Fw are used as the forward primer for the amplification of the nuclear and mitochondrial isoforms of *dut*, respectively. Rev‐1, Rev‐2, Rev‐3, and Rev‐4 are four reverse primer candidates for the amplification of both isoforms. PPIA‐Fw and PPIA‐Rev are used for the amplification of PPIA, while GAPDH‐Fw and GAPDH‐Rev are used for the amplification of GAPDH

Primer name	Sequence 5′‐3′	*T*_m_ [°C]
n*Dut*‐Fw	ATGCCCTGCTCGGAAGAT	64.8
m*Dut*‐Fw	CCGAGGAAGCCGAGGAG	66.3
Rev1	GCTCGAGCCCTCTTGGAG	65.3
Rev2	GCAGAGAAGCGCCATCCT	65.8
Rev3	GTGGCGTGCTCCGAGA	65.4
Rev4	TGTATAATCATAGGCACTGAATAGG	59.9
PPIA‐Fw	TGGCTATAAGGGTTCCTCCT	61.4
PPIA‐Rev	AAAACTGGGAACCGTTTGTG	63.6
GAPDH‐Fw	AGCTCATTTCCTGGTATGACAAT	62.5
GAPDH‐Rev	GGCCCCTCCTGTTATTATGG	64.1

We realized that the relatively high GC content of the first exon of the nuclear and the first two exons of the mitochondrial isoforms (70% for the nuclear and 75% for the mitochondrial isoform, respectively) may be associated with a complex secondary structure and accordingly a decrease in PCR efficiency 19. Hence, the secondary structure of the transcripts was analyzed with the OligoAnalyzer tool of Integrated DNA Technologies. The secondary structure of the PCR product with the isoform‐specific forward primers and Rev4 and Rev1 reverse primers is shown in Fig. S1. We selected the Rev4 and Rev1 reverse primers for this comparison since these will lead to the production of the longest and shortest PCR products. Using the Rev4 reverse primer, a longer PCR product is formed which results in a more complex secondary structure characterized with a higher melting temperature and greater change in Gibbs free energy as compared to the PCR product produced with Rev1 reverse primer (Fig. S1E).

For both nuclear and mitochondrial isoforms, all four candidate reverse primers were tested at a range of annealing temperature from 53.1 to 69 °C using a gradient thermal block. To test all primer pairs, the same amount of cDNA template was used in the PCR in order that the quantification cycle (C_q_) values of different primers can be compared for each target. Figure 1B shows the amplification curves for the mitochondrial isoform with the Rev1 reverse primer. Amplification associated with higher annealing temperature results in lower C_q_ value, indicating more efficient amplification. Melting curve analysis (Fig. 1C) and agarose gel electrophoresis (Fig. 1D) were performed to assess the specificity of the amplification reaction. In case of the PCR product for the mitochondrial isoform with the Rev1 reverse primer, the 78‐base pair (bp) length specific product is formed in the range of annealing temperature from 66 to 69 °C with a melting temperature of 89.5 °C; however, at lower temperature aspecific products appear (cf aspecific products with approximately 84 °C melting temperature on Fig. 1C). For the nuclear isoform with the Rev1 reverse primer, the image of agarose gel electrophoresis is shown in Fig. S2A. Between 55.4 and 69 °C, 53‐bp length specific product is formed. Figure S2B presents the agarose gel electrophoresis of the PCR products for both isoforms with Rev2 reverse primer in the same range of annealing temperature. For the nuclear isoform, the 73‐bp length specific product is formed from 58.6 to 69 °C, while for the mitochondrial isoform the 98‐bp specific product is formed only at 68 °C.

For comparison of the candidate reverse primers, the C_q_ values were plotted on the same diagram in the investigated temperature range (Fig. 1E,F for the mitochondrial and nuclear isoforms, respectively) also marking the specificity of the products. In case of the mitochondrial isoform, despite the specificity of the product with Rev4, the C_q_ values are much higher than in case of Rev1 and Rev2. Rev3 has a similar temperature range regarding the product specificity than Rev1 and Rev2; however, the C_q_ values are higher. Rev1 and Rev2 are quite similar to each other both in specificity and C_q_ values. In case of the nuclear isoform, all four reverse primers result in specific product formation from 62.7 to 69 °C. Rev1 and Rev2 are similar to each other regarding the C_q_ values, while Rev3 and Rev4 have higher values; thus, the use of Rev3 and Rev4 was ruled out. The differences in C_q_ values produced with the four reverse primer candidates may be the consequence of different amplicon length and corresponding PCR efficiencies, as amplification of shorter regions associated with less complex secondary structure proved to be more advantageous regarding the C_q_ values.

**Figure 2:**
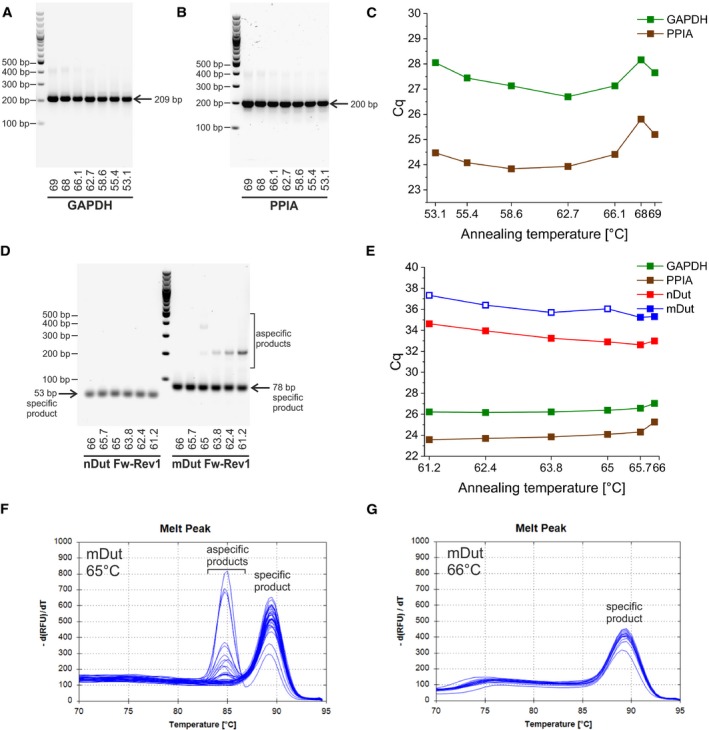
Determination of the optimal common annealing temperature with thermal gradient for the PCR amplification of both isoforms of the *Dut* transcript, GAPDH, and PPIA. (A, B) Agarose gel electrophoresis of the PCR products for GAPDH (A) and PPIA (B) at a range of annealing temperature from 53.1 to 69 °C. The specific product is indicated with an arrow. (C) Quantification cycles (C_q_) at a range of annealing temperatures from 53.1 to 69 °C for GAPDH and PPIA. (D) Agarose gel electrophoresis of the PCR products for both nuclear and mitochondrial isoforms with Rev1 reverse primer at a range of annealing temperature from 61.2 to 66 °C. The specific products are indicated with arrows. (E) Quantification cycles (C_q_) at a range of annealing temperatures from 61.2 to 66 °C with the selected primers. The solid squares indicate specific products as determined with agarose gel electrophoresis and melting curve analysis. Open squares indicate aspecific products. (F, G) Melting curve analysis of the PCR products for the mitochondrial isoform with the selected Rev1 reverse primer at 65 °C (F) and 66 °C (G) for the determination of product specificity

Besides the two isoforms of the *Dut* gene, two commonly used reference genes were also included in this study, glyceraldehyde 3‐phosphate dehydrogenase (GAPDH) and peptidyl‐prolyl cis‐trans isomerase A (PPIA) 23, 24, 25, 26, 27. The primers used for amplification of GAPDH and PPIA cDNAs produce specific products (209 and 200 bp for GAPDH and PPIA, respectively) in the whole range of annealing temperature from 53.1 to 69 °C (Fig. 2A,B); nonetheless, C_q_ values start to increase above 66 °C indicating suboptimal reaction conditions (Fig. 2C). We aimed to develop a method where transcripts from both nuclear and mitochondrial isoforms of the *Dut* gene as well as the two references (GAPDH and PPIA) can be analyzed on the same plate (i.e. gene maximization method). Thus, for determination of a common nearly optimal annealing temperature for all four targets, a detailed temperature gradient experiment was performed in the range from 61.2 to 66°C (Fig. 2D,E and Fig. S2C–E). With Rev1 reverse primer, the PCR product for the nuclear isoform was specific in the whole temperature range. For the mitochondrial isoform, however, specific product formation was observed only above 65 °C, while at 65 °C only a minuscule amount of aspecific product was found (Fig. 2D). With Rev2 reverse primer, aspecific product formation was found also at 65.7 °C and below for the mitochondrial isoform (Fig. S2C). For comparison of the candidate reverse primers in the detailed annealing temperature range, refer to Fig. S2D,E.

In conclusion, due to better specificity in case of the mitochondrial isoform, Rev1 was selected for the reverse primer. Fig. 2E shows the C_q_ values and specificity of the selected primers in the detailed annealing temperature range. For the C_q_ values and specificity of the selected primers in the broader 53.1‐69 °C annealing temperature range, refer to Fig. S2F. Both 65 and 66 °C could serve as a common annealing temperature for amplification of all four targets; however, at 66 °C, the C_q_ value for PPIA was increased by 1 cycle indicating suboptimal reaction. At both temperature points, several PCRs were performed to assess the product specificity (Fig. 2F,G). At 65 °C, some of the several PCR products were aspecific, while at 66 °C no aspecific product was found; thus, 66 °C was selected for the annealing temperature to be used.

#### Optimization of primer concentration

**Figure 3:**
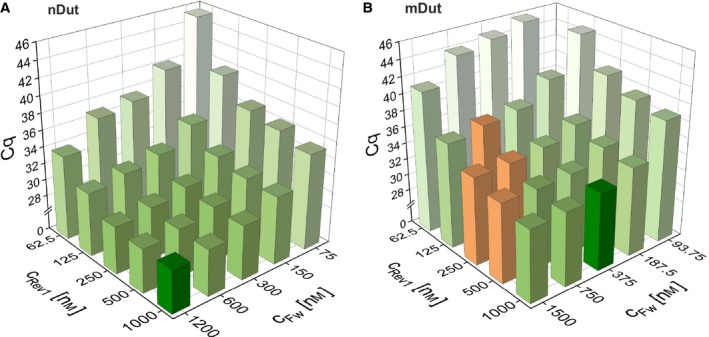
Determination of the optimal primer concentrations using concentration gradient of primers for both isoforms of the *Dut* transcript. (A) Quantification cycles as a function of the concentration of both the forward and reverse primers for the amplification of the nuclear isoform. Decreasing C_q_ values are indicated with increasing color intensity. The selected concentration point is shown with dark green. The C_q_ axis is broken from 2 to 28. (B) The same representation for the mitochondrial isoform as shown in panel A. Orange bars indicate aspecific product formation. Amplification at the least concentrated point of both primers did not occur; thus, no associated C_q_ value is shown in the graph

The importance of the optimization for primer concentrations is rarely taken into account; however, it can greatly influence the performance of the assay 28. Five point 2‐fold serial dilutions were prepared of each primer and a range of forward primer concentration was run against a range of reverse primer concentration in a form of a matrix for both nuclear and mitochondrial isoforms (Fig. 3) 29. For all reactions, a master mix was prepared with a small amount of cDNA that only lacked the primers to ensure constant concentration of all components of the reaction—except for the primers. Two technical replicates were used for each point of the matrix. In case of the nuclear isoform (Fig. 3A), higher concentrations of both primers resulted in lower C_q_ values, while all products were specific; therefore, the highest values of the tested concentrations were chosen for further experiments, that is 1200 nm for the forward and 1000 nm for the reverse primer. In case of the mitochondrial isoform (Fig. 3B), C_q_ values also decreased with higher concentrations; nonetheless, specificity of the product was compromised at the two highest concentration points of the forward primer. To avoid aspecific product formation, the third most concentrated point for the forward and the most concentrated point for the reverse primer was selected for further experiments, that is 375 nm for the forward primer and 1000 nm for the reverse primer.

#### Confirmation of the identity of the PCR products

**Figure 4:**
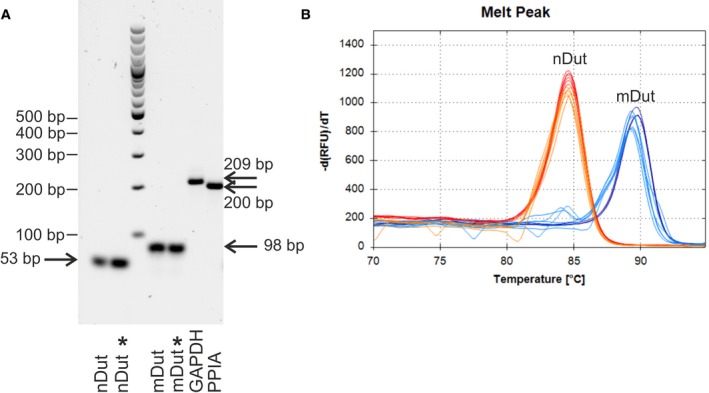
Determination of the specificity of the PCR products for both isoforms. (A) Agarose gel electrophoresis for both isoforms from one and two rounds of PCR and GAPDH and PPIA. Asterisks (*) indicate products from the second round of PCR. The specific products are indicated with arrows. (B) Melting curve analysis for both isoforms from one and two rounds of PCR. Red indicates the nuclear isoform from the single‐round PCR; orange indicates the second round. Dark blue indicates the mitochondrial isoform from the single‐round PCR; light blue indicates the second round

The GAPDH and PPIA products were sequenced; however, the products of the nuclear and mitochondrial dUTPase isoforms are too short for sequencing (53 and 78 bp, respectively); therefore, a two‐round amplification was performed (Fig. 4). Since for both isoforms, the Rev4 reverse primer had appropriate product specificity and length for sequencing (165 bp for the nuclear and 190 bp for the mitochondrial isoform), the first round of PCR was performed using ovary cDNA, Rev4 reverse primer and the appropriate forward primer, and then, the PCR products were purified and sent for sequencing. As the sequences of the products were confirmed, they were diluted and used as template in the second round of PCR with Rev1 reverse primer and the appropriate forward primer for both isoforms, since the PCR products with Rev4 reverse primer contain the whole sequence of the PCR products with Rev1 reverse primer. The second round PCR products were run on agarose gel along with products of a single‐round PCR using Rev1 reverse primer and appropriate forward primers for both isoforms and ovary cDNA (Fig. 4A). The melting curves of the second round and single‐round PCR products were also compared (Fig. 4B). Both comparisons confirmed the two products to be identical for both isoforms. Fig. 4A also shows representative bands of the PCR products for GAPDH and PPIA in the optimized PCR conditions.

#### Selection of a reverse transcription kit

**Figure 5:**
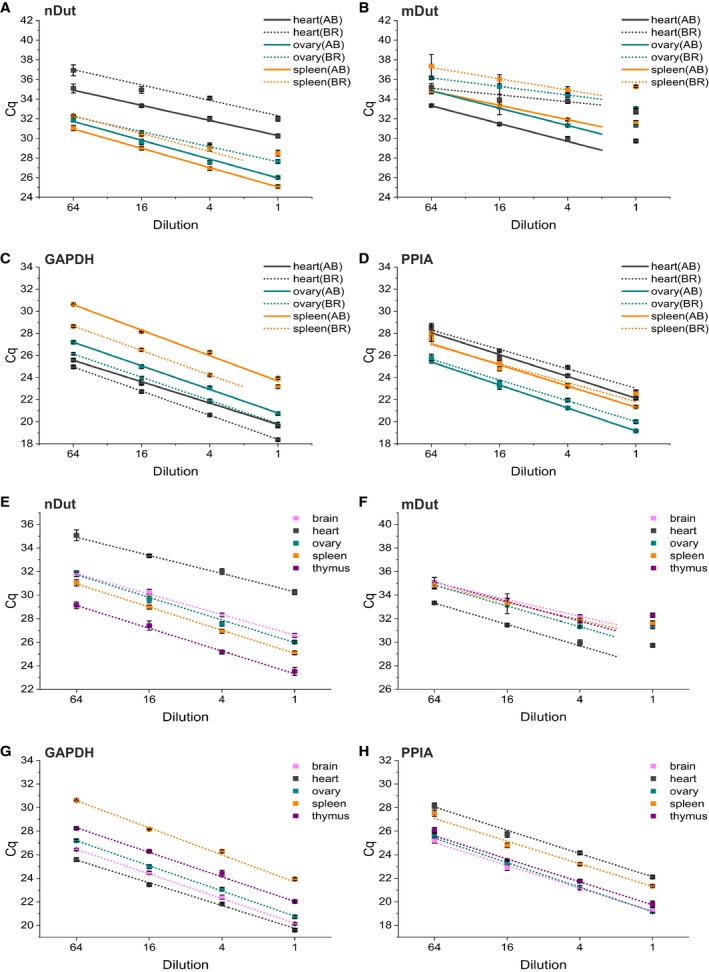
Selection of an appropriate cDNA synthesis kit for the reverse transcription reaction and determination of the linear concentration range. (A–D) Comparison of Applied Biosystems (AB) and Bio‐Rad (BR) cDNA synthesis kits for the nuclear (A) and mitochondrial (B) isoform, GAPDH (C), and PPIA (D) using heart, ovary, and spleen RNA samples. Fourfold serial dilutions with a starting concentration of 800 nm RNA solutions were introduced to cDNA synthesis followed by qPCR analysis. The points are calculated as the mean of three technical replicates. Error bars show standard deviation. In the linear concentration range, weighted least‐squares linear regression was performed. Solid lines correspond to the Applied Biosystems kit, while dotted lines correspond to the Bio‐Rad kit. (E–H) Fourfold serial dilutions with a starting concentration of 800 nm RNA solutions using five RNA samples derived from different organs introduced to the Applied Biosystems cDNA synthesis kit followed by qPCR analysis for the nuclear (E) and mitochondrial (F) isoforms, GAPDH (G) and PPIA (H). The points are calculated as the mean of three technical replicates. Error bars show standard deviation. In the linear concentration range, weighted least‐squares linear regression was performed.

Since the performance of the reverse transcription (RT) reaction heavily depends on the target concentration, total RNA concentration, priming strategy, the enzyme used and other parameters, careful selection and optimization of these criteria are of utmost importance 30, 31. First, we aimed to compare two commercially available RT kits. For this purpose, three different sources of RNA (heart, ovary, and spleen) were subjected to two different RT kits, High‐Capacity cDNA Reverse Transcription Kit (Applied Biosystems, Foster City, CA, USA) and iScript cDNA Synthesis Kit (Bio‐Rad, Hercules, CA, USA). Four point 4‐fold serial dilutions of RNA were prepared and introduced to the RT reaction with a starting amount of 800 ng (Fig. 5A–D). All cDNA samples were subjected to qPCR measurement with three technical replicates. For each serial dilution, weighted least‐squares linear regression was performed to the average of the three technical replicate C_q_ values. In case of the nuclear isoform and GAPDH, using the Applied Biosystems kit, the most concentrated point of the spleen sample was excluded because of lack of fit (Fig. 5A). In case of the mitochondrial isoform, fitting was performed using only three concentration points as the most concentrated points showed lack of fit for all samples (Fig. 5B). All fittings for the Applied Biosystems kit were appropriate with *R*‐square values above 0.99; however, in some cases the *R*‐square values were as low as 0.91 for the Bio‐Rad kit.

For both isoforms, the use of the High‐Capacity cDNA Reverse Transcription Kit (Applied Biosystems) resulted in considerably lower C_q_ values than iScript cDNA Synthesis Kit (Bio‐Rad) for all three RNA samples (Fig. 5A,B), indicating better performance of the RT reaction. In addition, the slopes of the regression lines were much lower for the mitochondrial isoform using the Bio‐Rad kit, indicating that the sensitivity of the assay could be compromised using the Bio‐Rad kit. For PPIA, the Applied Biosystems kit performs somewhat better, while in case of GAPDH, with Bio‐Rad kit lower C_q_ values could be achieved (Fig. 5C,D); however, both kits produced parallel linear lines. In conclusion, for both isoforms and PPIA, Applied Biosystems kit is more preferential and also applicable for GAPDH; thus, it was selected for further experiments.

#### Determination of the linear concentration range for the RT reaction

To provide comparability of the results of a RT‐qPCR study, the efficiency of the RT reaction must be kept constant. For this purpose, standardization of the reaction parameters—including total RNA concentration—and to work within the linear concentration range of the reaction is essential 30, 31. To determine the linear range of RNA concentration for the cDNA synthesis—thus ensuring lack of inhibition of the RT reaction in an extent that could compromise our results—five RNA samples from different organs (brain, heart, ovary, spleen, and thymus) were prepared (Fig. 5E–H). Four point 4‐fold serial dilutions of the samples were subjected to cDNA synthesis followed by qPCR measurement with three technical replicates. For each serial dilution, weighted least‐squares linear regression was performed to the average of the three technical replicate C_q_ values. In all cases, the most concentrated points fell out of the linear range for the mitochondrial isoform (Fig. 5F), whereas all concentration points were included in the linear range for the nuclear isoform (Fig. 5E), GAPDH (Fig. 5G), and PPIA (Fig. 5H). For further experiments, the second most concentrated point was selected; that is, 200 ng total RNA was subjected to cDNA synthesis to ensure linearity of the RT reaction for all four targets investigated.

#### Determination of the PCR efficiencies

In contrast to a commonly used method, we used serial dilutions of cDNA samples that were prepared with our verified RT reaction, instead of serial dilutions of standards, since the PCR efficiencies can be affected by components presented in the matrix. Thus, the use of serial dilutions of standards for the determination of PCR efficiencies inherently bears the possibility of the results being biased because of the different matrices 32.

**Figure 6:**
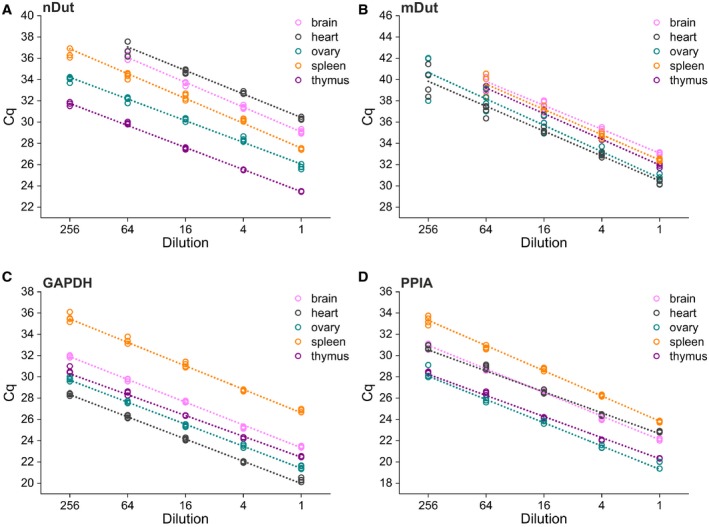
Evaluation of the amplification efficiency for both nuclear (A) and mitochondrial (B) isoforms of the *Dut* transcript, GAPDH (C), and PPIA (D). Fourfold serial dilutions of cDNA derived from 200 nm RNA solutions isolated from five different organs were subjected to qPCR measurement. All four technical replicates are shown, and weighted least‐squares linear regression was performed for each serial dilutions. In cases, where no amplification occurred for one or more technical replicates, all four values belonging to the concentration point were excluded from the analysis. For comparability of the slope of the fitted line, the range of the y‐axis on every graph is constant.

Five point 4‐fold serial dilutions were prepared from cDNA samples derived from five different organs (brain, heart, ovary, spleen, and thymus) and subjected to qPCR measurement with four technical replicates (Fig. 6). Weighted least‐squares linear regression was performed for all data points to counteract the effects of heteroscedasticity 33. However, in case of the two *Dut* isoforms, some replicates of the least concentrated point did not provide C_q_ values as amplification did not occur, in those cases the whole concentration point was excluded from the analysis (Fig. 6) 34. The intercept and slope of the curves and R‐square values were determined, and efficiency was calculated from the slope (Table S1) 35. Mean efficiency was calculated as the arithmetic mean of the individual efficiency values. The efficiency was estimated to be 88.4% for the nuclear isoform, 79.5% for the mitochondrial isoform, 94.1% for GAPDH, and 91.7% for PPIA. Generally, PCR efficiencies above 90% are accepted due to the assumption that precise and sensitive reactions are associated with high PCR efficiency 28. However, we demonstrate below that our assay is characterized with adequate performance parameters. For further experiments, the determined efficiencies were taken into account to calculate relative quantity values.

For all concentration points investigated, the reaction was linear. Thus, the dynamic range of the assay was linear for GAPDH between 20.3 and 35.5 C_q_, for PPIA between 19.7 and 33.3, for the nuclear isoform between 23.5 and 36.8, for the mitochondrial isoform between 30.3 and 40.6 C_q_ values. Moreover, the cDNA added to the reaction in a final dilution from 0.5 µL to 1.95 × 10^−4^ µL per 10 µL reaction resulted in no observable inhibition of the amplification. For further experiments, 0.31 µL of cDNA was added to each reaction.

### Expression levels of dUTPase isoforms in adult mouse tissues determined by the optimized RT‐qPCR protocol

To assess the applicability of the optimized RT‐qPCR assay, RNA was isolated from eight different organs of 10‐week‐old male and female mice. The integrity of all RNA samples was assessed with agarose gel electrophoresis (Fig. S3), and the purity was determined with NanoDrop (Table S2). Gel electrophoresis was our method of choice for the assessment of RNA integrity, because of its simplicity and affordability and considering that even moderately degraded RNA samples can be reliably quantified, provided the amplicons are short and expression is normalized to reference genes 36. All samples visualized on agarose gel showed the two distinct bands that were identified as the characteristic rRNA bands (28S and 18S) without any obvious degradation products or genomic DNA contamination. The 260/280 ratios were between 1.98 and 2.13 in most cases with two slightly higher values (2.22 and 2.61) (Table S2); thus, the purity of RNA was accepted for all samples 37.

**Figure 7:**
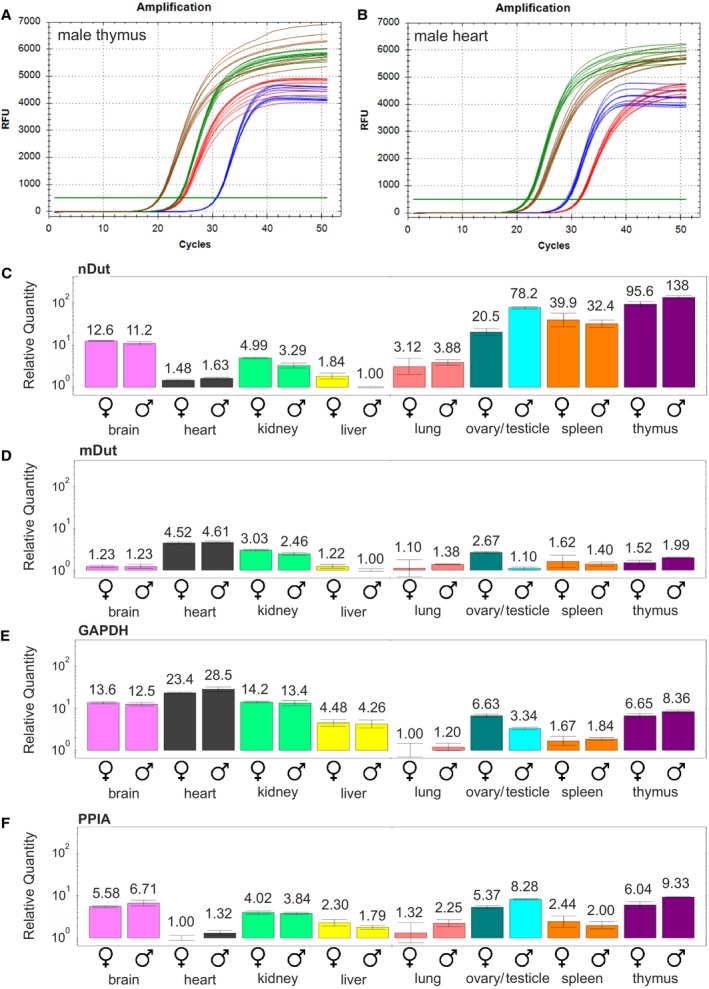
Evaluation of the method. (A, B) Amplification curves of male thymus (A) and male heart (B). Red color indicates the nuclear isoform, blue indicates the mitochondrial isoform, green indicates GAPDH, and brown indicates PPIA. Three biological replicates and three technical replicates for each biological replicate are shown for each graph. Threshold values are set to 500 RFU. RFU, relative fluorescent unit. (C–F) Relative quantity of gene expression of the nuclear isoform (C), the mitochondrial isoform (D), GAPDH (E) and PPIA (F) using ΔC_q_ method. In each case, logarithmic scales are used and the relative quantity of gene expression of the biological group with the lowest expression was set to 1. The biological groups of the same organs are juxtaposed with the female sex first. Error bars show standard deviation

Samples were subjected to the assay with three biological replicates from three different mice and three technical replicates for each biological replicate for each biological group (Fig. 7). In this study, biological groups are defined as different organs from either female or male mice. Fig. 7A,B shows the amplification curves of the four targets (the two dUTPase isoforms, and the two reference genes, GAPDH and PPIA) for organs with the highest level of expression of the nuclear and mitochondrial isoform, male thymus, and male heart, respectively. Graphs showing the amplification curves for all biological groups are included in Fig. S4.

We have observed that both the biological and technical scatter of the C_q_ values for each target within each biological group is considerably low as shown by the amplification curves (Fig. 7A,B and Fig. S4). The standard deviation of the C_q_ values between technical replicates was found to be 0.14, 0.13, 0.065, and 0.13 for the nuclear and mitochondrial isoform, GAPDH, and PPIA, respectively, as determined by ANOVA. Comparing the C_q_ values obtained for the different organs, we have also observed that the difference between the C_q_ values associated with the target genes GAPDH and PPIA showed considerably larger alterations than the C_q_ values for the mitochondrial dUTPase isoform. Therefore, based on the low variation of the C_q_ values within every biological group, we decided to use the ΔC_q_ method in Bio‐Rad cfx maestro software for a reliable assessment of the relative expression level of every biological group for all four targets (Fig. 7C–F). As no reference gene was used in this comparison and several qPCR runs were included, the threshold in the amplification plot was set to a common value to ensure comparability of the C_q_ values between qPCR runs. Although the use of the ΔC_q_ method with no reference genes may lead to errors due to the variation of RNA content and sample loading, the differences in the efficiency of RT and PCR and RNA integrity 23, 38, in our present experiments, the very low variation of the C_q_ values among the nine replicates indicated that such errors will not affect the reliability of our analysis. In fact, as shown in Fig. 7E,F, both reference genes showed considerably large variation among the different organs and the most stable expression pattern was shown by the mitochondrial dUTPase isoform (Fig. 7D).

Differences between the two sexes were small and found to be nonsignificant as determined by ANOVA (for the effect of sex, *P* values were 0.20, 0.94, 0.15, and 0.08 for the nuclear and mitochondrial isoform, GAPDH, and PPIA, respectively). However, larger differences were observed between organs that were highly significant (*P* < 10^−16^ for all four targets). In case of the nuclear isoform, thymus had the highest expression, followed by testicle, spleen, ovary, and brain, in order. Heart, kidney, liver, and lung showed markedly lower expression levels. The largest difference observed was between the expression levels of the nuclear dUTPase isoform in male liver and male thymus, with a 138‐fold increase in the latter. The expression level of the mitochondrial isoform was highest in the heart, followed by kidney and ovary. In contrast to the nuclear isoform, the mitochondrial isoform presented much smaller differences. An overall 4.61‐fold difference between the highest (male heart) and lowest (male liver) expression level was observed. As for GAPDH and PPIA, considerable differences exist between the organs, an overall 28.5‐fold difference in GAPDH expression and 9.33‐fold difference in PPIA expression was detected in comparing the highest expression to the lowest expression. The mitochondrial dUTPase isoform shows considerably stable expression pattern among various tissues, thus may be regarded as a possible reference gene for gene expression analysis involving several different organs with similar expression level of the mitochondrial isoform. In our study, heart, kidney, and ovary showed elevated expression levels; hence, normalization with the mitochondrial isoform for the comparison of gene expression between all these organs is not applicable, however may be appropriate for organs with similar expression levels.

## Conclusions

Our aim was to design, optimize, and validate a method for the simultaneous quantification of the expression levels of both nuclear and mitochondrial isoforms of dUTPase. These isoforms differ only in the first exon via alternative splicing. To develop an isoform‐specific method, amplification near the 5′ end of the transcripts was necessary, where the target sequences have a relatively high GC content and accordingly, a complex secondary structure that makes the optimization process rather difficult. In this paper, we introduced a detailed optimization procedure that could be useful for overcoming similar problems. Amplification of shorter regions associated with less complex secondary structure proved to be more advantageous. Since sequencing of short amplicons is not possible with the conventional Sanger sequencing method, we propose a two‐round amplification method for the confirmation of the identity of the PCR products. We found that higher primer concentration results in more efficient amplification as long as the product specificity is not compromised. We compared two commercially available reverse transcription kits for our purpose and realized that the selection of an RT kit determines the suitability of the RT‐qPCR assay fundamentally, and its applicability should be investigated for each target gene. We have successfully developed a method for the isoform‐specific determination of the expression of dUTPase taken into consideration the critical design parameters of the assay. In addition, we have evaluated the performance of the assay in terms of specificity, efficiency, precision, and the linear dynamic range. Our study is fully compliant with the MIQE guidelines (Table S3).

With regard to the expression of dUTPase isoforms among the different organs in adult mice, we observe that the nuclear isoform is expressed at a high level in thymus, spleen, and reproductive organs. It is well known that in both thymus and spleen, considerable mitosis occurs due to their involvement in lymphocyte production 39. Therefore, the biological role of these organs is in agreement with the well‐established role of dUTPase in dividing cells. Heart, liver, kidney, ovary, and brain are organs known to be rich in mitochondria 40, 41. However, elevated level of expression of the mitochondrial isoform of dUTPase was found only in heart, kidney, and ovary. Only a minuscule difference in the expression of the mitochondrial isoform was found in the other organs. In contrast, the difference in the expression levels of two commonly used reference genes, GAPDH and PPIA is much larger among the different mouse organs. This observation underlines the importance of the selection of appropriate reference genes in gene expression studies. Based on our results, we propose the use of the mitochondrial isoform of dUTPase as a potential reference gene for studies involving comparisons between different organs in mice. For gene expression studies involving mitochondrial proteins, normalization is usually carried out to expression levels of traditional reference genes 42, 43; however, the use of transcripts for mitochondrial proteins as reference genes, such as the mitochondrial isoform of dUTPase, may be beneficial for gene expression studies of mitochondrial proteins as well.

## Materials and methods

### Animals

Mice used in the experiments were produced and maintained in the Animal Care Facility at the NAIK Agricultural Biotechnology Center (FVB/N background, Envigo, Huntingdon, UK). Animals were housed in groups of 2–5 with free access to food and water. Animals were kept under standard light–dark cycle (06.00–18.00 h) at 22 °C. This study was carried out in strict accordance with the recommendations and rules in the Hungarian Code of Practice for the Care and Use of Animals for Scientific Purposes. The protocol was approved by the Animal Care and Ethics Committee of the NAIC‐Agricultural Biotechnology Institute and the Pest County’s governmental office (permission number: PEI/001/329‐4/2013). The method used for euthanasia: cervical dislocation. All efforts were made to minimize suffering.

### RNA isolation

Mice were euthanized and organs were prepared by macrodissection and were quick‐frozen in liquid nitrogen and stored in −70 °C until processing, which was carried out typically within a month. RNA isolation was carried out as quickly as possible using RNeasy Plus Mini Kit (Qiagen, Hilden, Germany) according to the manufacturer’s instructions. The organs were kept in liquid nitrogen, and approximately 20 mg of samples was excised. Before homogenization, samples were kept on ice. Mini beadbeater 96 (Biospec Products, Bartlesville, OK, USA) was used for homogenization with 0.9–2.0 mm RNase‐free stainless‐steel beads (Next Advance, Averill Park, NY, USA) for 1 min. DNase treatment was carried out with RNase‐Free DNase Set (Qiagen) according to the manufacturer’s instructions. RNA was eluted in 50 µL of RNase‐free water (Ambion, Austin, TX, USA), and the concentration and purity were determined with NanoDrop ND‐1000 (Table S2). Samples were diluted to 24 ng·µL^−1^ final concentration. The integrity and genomic DNA contamination of all RNA samples were tested using nondenaturing gel electrophoresis with 1% agarose gel in TBE buffer stained with ethidium bromide (Fig. S3). GeneRuler 1kb plus DNA ladder (Thermo Scientific, Waltham, MA, USA) was loaded as marker. Gel Doc XR+ Imager (Bio‐Rad) was used for imaging.

### cDNA synthesis

All RNA samples—except those that were used to assess the applicability of the iScript cDNA Synthesis Kit (Bio‐Rad)—were subjected to cDNA synthesis using the High‐Capacity cDNA Reverse Transcription Kit (Applied Biosystems) according to the manufacturer’s recommendations in reactions of 20 µL total volume with random hexamer primers. Thermal cycling conditions were set in ProFlex PCR System (Applied Biosystems). For the determination of optimal conditions for cDNA synthesis, four point 4‐fold serial dilutions of RNA were prepared and introduced to the RT reaction with a starting amount of 800 ng. For all other purposes, 200 ng of RNA was used for each individual cDNA synthesizing reaction, unless otherwise noted. cDNA samples were kept at −20 °C until further processing.

### Design of primers

For the design of primers, the sequences of the transcripts of interest were downloaded from the RefSeq database. The gene *Dut* has two known splice variants that differ in the first exons (accession numbers: NM_023595.6 and NM_001159646.1, for the nuclear and mitochondrial isoform, respectively). For our experiments, we have confirmed the corresponding genomic DNA sequence of *Dut* by sequencing and isoform‐specific primers were designed. Two reference genes were also used, GAPDH and PPIA. GAPDH has two known splice variants in mice (accession numbers: NM_001289726.1 and NM_008084.3) differing in the first exons; thus, primers were designed to amplify the 3′ common region of the mRNA. For GAPDH, an exon junction spanning forward and an exonic reverse primer were designed. PPIA has only one splice variant (NM_008907.2). For PPIA, exonic forward and reverse primers were designed. The design of primers and the prediction of the specificity were carried out with the Primer‐BLAST tool 22. The oligonucleotides were ordered from Sigma–Aldrich (St. Louis, MO, USA) with desalting purification.

### qPCR method

For the amplification, MyTaq HS Mix (Bioline, London, UK), Evagreen dye (Biotium, Hayward, CA, USA), RNase‐free water (Ambion), cDNA template, and appropriate primers were mixed in 10 µL final volume reactions. Clear Hard‐Shell 96‐Well PCR Plates (Bio‐Rad) and Microseal 'B' PCR Plate Sealing Film (Bio‐Rad) were used.

For the temperature gradient optimization, cDNA reverse‐transcribed from 200 ng of RNA isolated from 10‐week‐old ovary sample was used to result in a final dilution of 0.05 µL/10 µL reaction. Final dilution in these cases was reached in a series of dilutions. For the primer concentration optimization, cDNA reverse‐transcribed from 200 ng of RNA isolated from 10‐week‐old ovary sample was used to result in a final dilution of 0.055 µL/10 µL reaction. For the determination of optimal conditions for cDNA synthesis, RNA was isolated from 10‐week‐old female brain, heart, and ovary and 4‐week‐old male spleen and thymus with three biological replicates from different mice for each organ. Equal amount of each replicate RNA sample was pooled and cDNA was prepared—with either High‐Capacity cDNA Reverse Transcription Kit (Applied Biosystems) or iScript cDNA Synthesis Kit (Bio‐Rad)—using 800, 200, 50, and 12.5 ng of total RNA. The resulting cDNA samples were subjected to qPCR in a final dilution of 0.31 µL/10 µL reaction.

For determining the PCR efficiencies, cDNA samples with three biological replicates from different mice for each organ were reverse‐transcribed from 200 ng of RNA isolated from 10‐week‐old female brain, heart, and ovary and 4‐week‐old male spleen and thymus. Equal amount of each replicate cDNA sample was pooled and used to prepare fourfold serial dilutions. cDNA was subjected to qPCR in final dilutions of 0.5 µL, 0.0125 µL, 3125 × 10^−3^ µL, 7.81 × 10^−4^, and 1.95 × 10^−4^ µL per 10 µL reaction.

For the assessment of the optimized RT‐qPCR assay, cDNA from 200 ng of RNA isolated from 8 different organs (brain, heart, kidney, liver, lung, ovary/testicle, spleen, and thymus) of 10‐week‐old male and female mice were subjected to qPCR in a final dilution of 0.31 µL/10 µL reaction. Each organ from either female or male mice forms a biological group, which includes three biological replicates from different mice.

The primers used for amplification are summarized in Table 1. GAPDH‐Fw and GAPDH‐Rev primers were used in a final concentration of 315 nm; PPIA‐Fw and PPIA‐Rev primers were used in a final concentration of 425 and 500 nm, respectively. For the temperature gradient optimization, Rev1, Rev2, Rev3, and Rev4 primers were used in a final concentration of 250 nm. For the primer concentration optimization, Rev1, Rev2, Rev3, and Rev4 primers were used in final concentrations of 1000, 500, 250, 125, and 62.5 nm. For all other experiments, 1000 nm final concentration was used for Rev1 primer. For the temperature gradient optimization, n*Dut*‐Fw and m*Dut*‐Fw primers were used in final concentrations of 300 and 375 nm, respectively. For the primer concentration optimization, n*Dut*‐Fw primer were used in final concentrations of 1200, 600, 300, 250, and 125 nm, while m*Dut*‐Fw primer was used in final concentrations of 1500, 750, 375, 187.7, and 93.75 nm. For all other experiments, 1200 nm final concentration was used for n*Dut*‐Fw primer and 375 nm final concentration was used for m*Dut*‐Fw primer.

A gene maximizing plate design was used with manual reaction setup. Each plate contained four replicates of no template controls (NTC) for each target and three technical replicates of no reverse transcriptase (NRT) controls for each target and each sample. All other reactions were carried out in triplicates, unless otherwise noted.

PCR cycling was performed at 95 °C for 5 min followed by 50 cycles of denaturation at 95 °C for 30 s, annealing and extension at 66 °C for 30 s, and melting curve analysis from 60 to 95 °C at a rate of 0.5 °C/5 s. CFX96 real‐time PCR detection system (Bio‐Rad) was used for thermal cycling and detection.

### Assessment of the specificity of PCR

The specificity of the PCR products was tested first using gel electrophoresis with 2% agarose gel in TBE buffer stained with GelRed nucleic acid gel stain (Biotium). GeneRuler 100 bp plus DNA ladder (Thermo Scientific) was loaded as marker. Gel Doc XR+ Imager (Bio‐Rad) was used for imaging. To confirm the identity of all PCR products—except for the PCR product for the nuclear and mitochondrial isoforms, which were too short for sequencing—DNA was isolated from agarose gel with NucleoSpin gel and PCR clean‐up (Macherey‐Nagel, Duren, Germany) according to the manufacturer’s recommendations and sequenced by Microsynth Seqlab, Göttingen, Germany. To confirm the identity of the PCR products for the nuclear and mitochondrial isoforms, PCR product for both isoforms with Rev4 reverse primer using ovary cDNA was isolated from agarose gel. The concentration of the DNA was determined with NanoDrop ND‐2000, and the samples were used as template in a second round of PCR with 10 and 0.1 fg·µL^−1^ final concentration for both isoforms. For further reactions, the specificity of the formed PCR products was confirmed with melting curve analysis. Results were accepted only with uncompromised product specificity—or indicated otherwise for the process of optimization.

### Analysis of data

Amplification curves and melting curves were illustrated by cfx maestro 1.1 software (Bio‐Rad). C_q_ determination mode was set to single threshold. For primer optimization experiments, all data were used in the analysis, and none were excluded. However, in other experiments we have set several criteria to define outlier data. First, C_q_ values were identified as outlier and disposed of, if the final RFU value was lower by 10% than the average final RFU value for the corresponding target. Second, C_q_ values were identified as outlier and disposed of, if the melting curve analysis showed aspecific product formation. Approximately 5% of the data were excluded from the analysis based on these two criteria. Third, the differences in C_q_ values between the unknown samples and NTC/NRT controls were investigated. In every case, the difference was more than 5 cycles; however, in most cases, it was more than 8 cycles, thus all results were accepted based on this criterion. Relative quantity was calculated and illustrated by CFX Maestro software (Bio‐Rad). Scatter graphs and 3D bar graphs were created with originpro 2018 (OriginLab Corp., Northampton, MA, USA).

For the results presented in Fig. 7, ANOVA was performed with tibco statistica 13.4 (Tibco Software Inc., Palo Alto, CA, USA). For the model, the random ‘sample’ variable was nested into the interaction term of the variables ‘organ’ and ‘sex’, and type III (orthogonal) decomposition was used. To check for the homogeneity of variances, the Bartlett chi‐square test was used, and normality of the within‐cell residuals was checked on normal probability plots. The residuals were also checked by plotting them against the observed values. In all cases, the assumptions for the use of ANOVA were met. The standard deviation of the C_q_ values was calculated as the square root of the mean sum of squares due to error.

## Conflict of interest

The authors declare no conflict of interest.

## Author contributions

GR and BV planned the experiments; GR and NN performed the experiments and analyzed the data; ZG, TP, and LH were involved in sample preparation; GR, NN, and BV wrote the paper.

## Supporting information


**Fig. S1. **Possible secondary structures of the PCR products as predicted by the OligoAnalyzer tool of Integrated DNA Technologies. The introduced structures were selected based on the highest change in Gibbs free energy according to the tool. Blue circles indicate A‐T interaction, red circles indicate G‐C interaction, green indicates G‐T interaction. (A, B) Secondary structure of the nuclear (A) and mitochondrial (B) isoform with isoform‐specific forward primers and Rev4 reverse primer. (C, D) Secondary structure of the nuclear (C) and mitochondrial (D) isoform with isoform‐specific forward primers and Rev1 reverse primer. (E) Table summarising the thermodynamic parameters of the four introduced structures.
**Fig. S2. **(A) Agarose gel electrophoresis of the PCR products for the nuclear isoform with Rev1 reverse primer at a range of annealing temperature from 53.1 to 69 °C. The specific product is indicated with an arrow. Vertical line indicates cropping of the image. (B, C) Agarose gel electrophoresis of the PCR products for both nuclear and mitochondrial isoforms with Rev2 reverse primer at a range of annealing temperature from 53.1 to 69 °C (B) and from 61.2 to 66 °C (C). The specific products are indicated with arrows. Vertical line indicates cropping of the image. (D, E) Quantification cycles (Cq) at a range of annealing temperatures from 61.2 to 66 °C with all four reverse primer candidates for the mitochondrial isoform (D) and nuclear isoform (E). The solid squares indicate specific products as determined with agarose gel electophoresis and melting curve analysis. Open squares indicate aspecific products. (F) Quantification cycles (Cq) at a range of annealing temperatures from 53.1 to 69 °C with the selected primers. The solid squares indicate specific products as determined with agarose gel electophoresis and melting curve analysis. Open squares indicate aspecific products.
**Fig. S3. **Agarose gel electrophoresis of the RNA samples used in this study. Vertical lines indicate cropping of the image.
**Fig. S4. **Amplification curves of all the biological groups. Red colour indicates the nuclear isoform, blue indicates the mitochondrial isoform, green indicates GAPDH and brown indicates PPIA. Three biological replicates and three technical replicates for each biological replicates are shown for each graph. Threshold values are set to 500 RFU. RFU, relative fluorescent unit. The biological groups of the same organs are juxtaposed with the female sex first.
**Table S1** Parameters of the fitted linear curves used to determine qPCR efficiencies. Weighted least squares linear regression was performed to the Cq values from 4‐fold cDNA dilution. Accordingly, base 4 logarithm was used for the efficiency calculation.
**Table S2** Purity of the RNA samples used in this study indicated by 260/280 absorbance ratio values measured by Nanodrop.
**Table S3** MIQE checklist for our study. ✓: fulfillment of the requirements; – : not provided data; N/A : not applicable parameter.

## References

[1] Vértessy BG and Tóth J (2009) Keeping uracil out of DNA: physiological role, structure and catalytic mechanism of dUTPases. Acc Chem Res 42, 97–106. 18837522 10.1021/ar800114w PMC2732909

[2] Kerepesi C , Szabó JE , Papp‐Kádár V , Dobay O , Szabó D , Grolmusz V and Vértessy BG (2016) Life without dUTPase. Front Microbiol 7, 1768. 27933035 10.3389/fmicb.2016.01768 PMC5122711

[3] Dengg M , Garcia‐Muse T , Gill SG , Ashcroft N , Boulton SJ and Nilsen H (2006) Abrogation of the CLK‐2 checkpoint leads to tolerance to base‐excision repair intermediates. EMBO Rep 7, 1046–1051. 16964178 10.1038/sj.embor.7400782 PMC1618380

[4] Kumar H , Kehrer J , Singer M , Reinig M , Santos JM , Mair GR and Frischknecht F (2019) Functional genetic evaluation of DNA house‐cleaning enzymes in the malaria parasite: dUTPase and Ap4AH are essential in *Plasmodium berghei* but ITPase and NDH are dispensable. Expert Opin Ther Targets 23, 251–261. 30700216 10.1080/14728222.2019.1575810

[5] Muha V , Horváth A , Békési A , Pukáncsik M , Hodoscsek B , Merényi G , Róna G , Batki J , Kiss I , Jankovics F *et al* (2012) Uracil‐containing DNA in Drosophila: stability, stage‐specific accumulation, and developmental involvement. PLoS Genet 8, e1002738. 22685418 10.1371/journal.pgen.1002738 PMC3369950

[6] Muha V , Zagyva I , Venkei Z , Szabad J and Vértessy BG (2009) Nuclear localization signal‐dependent and ‐independent movements of *Drosophila melanogaster* dUTPase isoforms during nuclear cleavage. Biochem Biophys Res Commun 381, 271–275. 19232319 10.1016/j.bbrc.2009.02.036

[7] Ladner RD and Caradonna SJ (1997) The human dUTPase gene encodes both nuclear and mitochondrial isoforms. Differential expression of the isoforms and characterization of a cDNA encoding the mitochondrial species. J Biol Chem 272, 19072–19080. 9228092 10.1074/jbc.272.30.19072

[8] Róna G , Marfori M , Borsos M , Scheer I , Takács E , Tóth J , Babos F , Magyar A , Erdei A , Bozóky Z *et al* (2013) Phosphorylation adjacent to the nuclear localization signal of human dUTPase abolishes nuclear import: structural and mechanistic insights. Acta Crystallogr D Biol Crystallogr 69, 2495–2505. 24311590 10.1107/S0907444913023354

[9] Pri‐Hadash A , Hareven D and Lifschitz E (1992) A meristem‐related gene from tomato encodes a dUTPase: analysis of expression in vegetative and floral meristems. Plant Cell 4, 149–159. 1321683 10.1105/tpc.4.2.149 PMC160116

[10] Békési A , Zagyva I , Hunyadi‐Gulyás E , Pongrácz V , Kovári J , Nagy AO , Erdei A , Medzihradszky KF and Vértessy BG (2004) Developmental regulation of dUTPase in Drosophila melanogaster. J Biol Chem 279, 22362–22370. 14996835 10.1074/jbc.M313647200

[11] Wilson PM , Fazzone W , LaBonte MJ , Deng J , Neamati N and Ladner RD (2008) Novel opportunities for thymidylate metabolism as a therapeutic target. Mol Cancer Ther 7, 3029–3037. 18790783 10.1158/1535-7163.MCT-08-0280 PMC2597111

[12] Takatori H , Yamashita T , Honda M , Nishino R , Arai K , Yamashita T , Takamura H , Ohta T , Zen Y and Kaneko S (2010) dUTP pyrophosphatase expression correlates with a poor prognosis in hepatocellular carcinoma. Liver Int 30, 438–446. 19968781 10.1111/j.1478-3231.2009.02177.x

[13] Kawahara A , Akagi Y , Hattori S , Mizobe T , Shirouzu K , Ono M , Yanagawa T , Kuwano M and Kage M (2009) Higher expression of deoxyuridine triphosphatase (dUTPase) may predict the metastasis potential of colorectal cancer. J Clin Pathol 62, 364–369. 19052026 10.1136/jcp.2008.060004 PMC2656677

[14] Huntley MA , Lou M , Goldstein LD , Lawrence M , Dijkgraaf GJP , Kaminker JS and Gentleman R (2016) Complex regulation of ADAR‐mediated RNA‐editing across tissues. BMC Genom 17, 61. 10.1186/s12864-015-2291-9 PMC4714477 26768488

[15] Brawand D , Soumillon M , Necsulea A , Julien P , Csárdi G , Harrigan P , Weier M , Liechti A , Aximu‐Petri A , Kircher M *et al* (2011) The evolution of gene expression levels in mammalian organs. Nature 478, 343–348. 22012392 10.1038/nature10532

[16] Keane TM , Goodstadt L , Danecek P , White MA , Wong K , Yalcin B , Heger A , Agam A , Slater G , Goodson M *et al* (2011) Mouse genomic variation and its effect on phenotypes and gene regulation. Nature 477, 289–294. 21921910 10.1038/nature10413 PMC3276836

[17] Soumillon M , Necsulea A , Weier M , Brawand D , Zhang X , Gu H , Barthès P , Kokkinaki M , Nef S , Gnirke A *et al.* (2013) Cellular source and mechanisms of high transcriptome complexity in the mammalian testis. Cell Rep 3, 2179–2190. 23791531 10.1016/j.celrep.2013.05.031

[18] Barbosa‐Morais NL , Irimia M , Pan Q , Xiong HY , Gueroussov S , Lee LJ , Slobodeniuc V , Kutter C , Watt S , Colak R *et al* (2012) The evolutionary landscape of alternative splicing in vertebrate species. Science 338, 1587–1593. 23258890 10.1126/science.1230612

[19] Mamedov TG , Pienaar E , Whitney SE , TerMaat JR , Carvill G , Goliath R , Subramanian A and Viljoen HJ (2008) A fundamental study of the PCR amplification of GC‐rich DNA templates. Comput Biol Chem 32, 452–457. 18760969 10.1016/j.compbiolchem.2008.07.021 PMC2727727

[20] Bustin SA , Benes V , Garson JA , Hellemans J , Huggett J , Kubista M , Mueller R , Nolan T , Pfaffl MW , Shipley GL *et al* (2009) The MIQE guidelines: minimum information for publication of quantitative real‐time PCR experiments. Clin Chem 55, 611–622. 19246619 10.1373/clinchem.2008.112797

[21] Debode F , Marien A , Janssen É , Bragard C and Berben G (2017) The influence of amplicon length on real‐time PCR results. Biotechnol Agron Soc Environ 21, 3–11.

[22] Ye J , Coulouris G , Zaretskaya I , Cutcutache I , Rozen S and Madden TL (2012) Primer‐BLAST: a tool to design target‐specific primers for polymerase chain reaction. BMC Bioinformatics 13, 134. 22708584 10.1186/1471-2105-13-134 PMC3412702

[23] Radonić A , Thulke S , Mackay IM , Landt O , Siegert W and Nitsche A (2004) Guideline to reference gene selection for quantitative real‐time PCR. Biochem Biophys Res Commun 313, 856–862. 14706621 10.1016/j.bbrc.2003.11.177

[24] Gubern C , Hurtado O , Rodríguez R , Morales JR , Romera VG , Moro MA , Lizasoain I , Serena J and Mallolas J (2009) Validation of housekeeping genes for quantitative real‐time PCR in in‐vivo and in‐vitro models of cerebral ischaemia. BMC Mol Biol 10, 57. 19531214 10.1186/1471-2199-10-57 PMC2706836

[25] Ren S , Zhang F , Li C , Jia C , Li S , Xi H , Zhang H , Yang L and Wang Y (2010) Selection of housekeeping genes for use in quantitative reverse transcription PCR assays on the murine cornea. Mol Vis 16, 1076–1086. 20596249 PMC2893048

[26] Ali H , Du Z , Li X , Yang Q , Zhang YC , Wu M , Li Y and Zhang G (2015) Identification of suitable reference genes for gene expression studies using quantitative polymerase chain reaction in lung cancer in vitro. Mol Med Rep 11, 3767–3773. 25573171 10.3892/mmr.2015.3159

[27] Gong Z‐K , Wang S‐J , Huang Y‐Q , Zhao R‐Q , Zhu Q‐F and Lin W‐Z (2014) Identification and validation of suitable reference genes for RT‐qPCR analysis in mouse testis development. Mol Genet Genomics 289, 1157–1169. 24952483 10.1007/s00438-014-0877-6

[28] Nolan T , Hands RE and Bustin SA (2006) Quantification of mRNA using real‐time RT‐PCR. Nat Protoc 1, 1559–1582. 17406449 10.1038/nprot.2006.236

[29] Gunson R , Gillespie G and Carman WF (2003) Optimisation of PCR reactions using primer chessboarding. J Clin Virol 26, 369–373. 12637087 10.1016/s1386-6532(03)00006-4

[30] Ståhlberg A , Håkansson J , Xian X , Semb H and Kubista M (2004) Properties of the reverse transcription reaction in mRNA quantification. Clin Chem 50, 509–515. 14726469 10.1373/clinchem.2003.026161

[31] Miranda JA and Steward GF (2017) Variables influencing the efficiency and interpretation of reverse transcription quantitative PCR (RT‐qPCR): An empirical study using Bacteriophage MS2. J Virol Methods 241, 1–10. 27940257 10.1016/j.jviromet.2016.12.002

[32] Pfaffl MW (2004) Quantification strategies in real‐time PCR In A‐Z of Quantitative PCR (Bustin SA, ed.), pp. 87–112. International University Line, La Jolla, CA.

[33] Almeida AM , Castel‐Branco MM and Falcão AC (2002) Linear regression for calibration lines revisited: weighting schemes for bioanalytical methods. J Chromatogr B Analyt Technol Biomed Life Sci 774, 215–222. 10.1016/s1570-0232(02)00244-1 12076691

[34] Ellison SLR , English CA , Burns MJ and Keer JT (2006) Routes to improving the reliability of low level DNA analysis using real‐time PCR. BMC Biotechnol 6, 33. 16824215 10.1186/1472-6750-6-33 PMC1559608

[35] Svec D , Tichopad A , Novosadova V , Pfaffl MW and Kubista M (2015) How good is a PCR efficiency estimate: recommendations for precise and robust qPCR efficiency assessments. Biomol Detect Quantif 3, 9–16. 27077029 10.1016/j.bdq.2015.01.005 PMC4822216

[36] Fleige S and Pfaffl MW (2006) RNA integrity and the effect on the real‐time qRT‐PCR performance. Mol Aspects Med 27, 126–139. 16469371 10.1016/j.mam.2005.12.003

[37] Bustin SA and Nolan T (2004) Template handling, preparation, and quantification In A‐Z of Quantitative PCR (Bustin S, ed.), pp. 141–215. International University Line, 2004–2006, La Jolla, CA.

[38] Hruz T , Wyss M , Docquier M , Pfaffl MW , Masanetz S , Borghi L , Verbrugghe P , Kalaydjieva L , Bleuler S , Laule O *et al.* (2011) RefGenes: identification of reliable and condition specific reference genes for RT‐qPCR data normalization. BMC Genom 12, 156. 10.1186/1471-2164-12-156 PMC3072958 21418615

[39] Alberts B , Johnson A , Lewis J , Morgan D , Raff M , Roberts K and Peter W (2015) The innate and adaptive immune systems In Molecular Biology of the Cell, 6th edn (Zayatz E-B, Lewis SG and Clayton J, eds), pp. 1297–1342. Garland Science, New York, NY.

[40] Veltri KL , Espiritu M and Singh G (1990) Distinct genomic copy number in mitochondria of different mammalian organs. J Cell Physiol 143, 160–164. 2318903 10.1002/jcp.1041430122

[41] Bromfield JJ and Piersanti RL (2019) In Mammalian oogenesis: the fragile foundation of the next generation In The Ovary, 3rd edn (Leung PCK and Adashi EY, eds), pp. 157–205. Academic Press, Cambridge, MA.

[42] Tang P‐A , Duan J‐Y , Wu H‐J , Ju X‐R and Yuan M‐L (2017) Reference gene selection to determine differences in mitochondrial gene expressions in phosphine‐susceptible and phosphine‐resistant strains of *Cryptolestes ferrugineus*, using qRT‐PCR. Sci Rep 7, 7047. 28765619 10.1038/s41598-017-07430-2 PMC5539111

[43] Merlo Pich M , Raule N , Catani L , Fagioli ME , Faenza I , Cocco L and Lenaz G (2004) Increased transcription of mitochondrial genes for Complex I in human platelets during ageing. FEBS Lett 558, 19–22. 14759509 10.1016/S0014-5793(03)01520-5

