# Supplementary material for: Evaluation of critical design parameters for RT‐qPCR‐based analysis of multiple dUTPase isoform genes in mice
Source: FEBS Open Bio. 2019 May 29;9(6):1153–70. doi: 10.1002/2211-5463.12654 (PMC6551494; doi:10.1002/2211-5463.12654)
Supplement: Supplementary file 1 — Fig. S1. Possible secondary structures of the PCR products as predicted by the OligoAnalyzer tool of Integrated DNA Technologies. The introduced structures were selected based on the highest change in Gibbs free energy according to the tool. Blue circles indicate A‐T interaction, red circles indicate G‐C interaction, green indicates G‐T interaction. (A, B) Secondary structure of the nuclear (A) and mitochondrial (B) isoform with isoform‐specific forward primers and Rev4 reverse primer. (C, D) Secondary structure of the nuclear (C) and mitochondrial (D) isoform with isoform‐specific forward primers and Rev1 reverse primer. (E) Table summarising the thermodynamic parameters of the four introduced structures. Fig. S2. (A) Agarose gel electrophoresis of the PCR products for the nuclear isoform with Rev1 reverse primer at a range of annealing temperature from 53.1 to 69 °C. The specific product is indicated with an arrow. Vertical line indicates cropping of the image. (B, C) Agarose gel electrophoresis of the PCR products for both nuclear and mitochondrial isoforms with Rev2 reverse primer at a range of annealing temperature from 53.1 to 69 °C (B) and from 61.2 to 66 °C (C). The specific products are indicated with arrows. Vertical line indicates cropping of the image. (D, E) Quantification cycles (Cq) at a range of annealing temperatures from 61.2 to 66 °C with all four reverse primer candidates for the mitochondrial isoform (D) and nuclear isoform (E). The solid squares indicate specific products as determined with agarose gel electophoresis and melting curve analysis. Open squares indicate aspecific products. (F) Quantification cycles (Cq) at a range of annealing temperatures from 53.1 to 69 °C with the selected primers. The solid squares indicate specific products as determined with agarose gel electophoresis and melting curve analysis. Open squares indicate aspecific products. Fig. S3. Agarose gel electrophoresis of the RNA samples used in this study. Vertical lines [file FEB4-9-1153-s001.pdf]

Supplementary Information

Evaluation of critical design parameters for the RT-qPCR based analysis of dUTPase gene expression involving multiple isoforms in mice

Gergely Attila Rácz[1,2*], Nikolett Nagy[1,2], Zoltán Gál[3,4], Tímea Pintér[3], László Hiripi[3], Beáta G. Vértessy[1,2*]

[1]Institute of Enzymology, RCNS, Hungarian Academy of Sciences, Budapest, Hungary

[2]Department of Applied Biotechnology and Food Sciences, Budapest University of Technology and Economics, Budapest, Hungary

[3]Department of Animal Biotechnology, Agricultural Biotechnology Institute, National Agricultural Research and Innovation Centre, Gödöllő, Hungary

[4]Faculty of Agricultural and Environmental Science, Szent István University, Gödöllő, Hungary

[*]corresponding authors

Correspondance

Gergely Attila Rácz and Beáta G. Vértessy, Department of Applied Biotechnology and Food Science, Budapest University of Technology and Economics, Műegyetem rkp. 3, H-1111 Budapest, Hungary

Tel.: +36 1 4633854

E-mails: racz.gergely@ttk.mta.hu, vertessy@mail.bme.hu

Supplementary Tables

Supplementary Table 1. Parameters of the fitted linear curves used to determine qPCR efficiencies. Weighted least squares linear regression was performed to the C_q values from 4-fold cDNA dilution. Accordingly, base 4 logarithm was used for the efficiency calculation.

Target	Parameter	Heart	Thymus	Ovary	Brain	Spleen	Mean efficiency (%)
**n*Dut***	Intercept	30.4	23.5	26.0	29.1	27.6	**88.1%**
	Slope	-2.212	-2.074	-2.041	-2.330	-2.333	
	Efficiency	0.872	0.951	0.972	0.813	0.811	
	R^2	0.996	0.999	0.995	0.997	0.993	
**m*Dut***	Intercept	30.5	32.0	30.7	33.1	32.4	**79.5%**
	Slope	-2.341	-2.416	-2.481	-2.249	-2.381	
	Efficiency	0.808	0.775	0.748	0.852	0.790	
	R^2	0.996	0.999	1.000	1.000	1.000	
**GAPDH**	Intercept	20.0	22.4	21.4	23.3	26.6	**94.1%**
	Slope	-2.089	-1.961	-2.073	-2.140	-2.208	
	Efficiency	0.942	1.028	0.952	0.911	0.874	
	R^2	0.999	0.999	0.999	0.998	0.997	
**PPIA**	Intercept	22.6	20.3	19.3	22.1	23.8	**91.7%**
	Slope	-1.970	-1.984	-2.186	-2.215	-2.369	
	Efficiency	1.021	1.012	0.885	0.870	0.795	
	R^2	0.988	0.998	0.996	0.998	0.999	

Supplementary Table 2. Purity of the RNA samples used in this study indicated by 260/280 absorbance ratio values measured by Nanodrop.

Organ	Sex	No.	260/280
brain	female	1	2.13
		2	2.02
		3	2.11
	male	1	2.08
		2	2.10
		3	2.13
heart	female	1	2.09
		2	2.10
		3	2.08
	male	1	2.03
		2	2.02
		3	1.98
kidney	female	1	2.11
		2	2.09
		3	2.09
	male	1	2.06
		2	2.07
		3	2.07
liver	female	1	2.10
		2	2.10
		3	2.09
	male	1	2.10
		2	2.11
		3	2.07
lung	female	1	2.61
		2	2.06
		3	2.05
	male	1	2.03
		2	2.12
		3	2.22
ovary	female	1	2.09
		2	2.11
		3	2.07
testicle	male	1	2.12
		2	2.08
		3	2.11
spleen	female	1	2.08
		2	2.06
		3	2.04
	male	1	2.05
		2	2.07
		3	2.05
thymus	female	1	2.08
		2	2.07
		3	2.07
	male	1	2.05
		2	2.06
		3	2.12

Supplementary Table 3. MIQE checklist for our study. ✓: fulfillment of the requirements; – : not provided data; N/A : not applicable parameter.

Item to check	Importance	Checklist
**Experimental design**		
Definition of experimental and control groups	E	✓
Number within each group	E	✓
Assay carried out by the core or investigator's laboratory?	D	✓
Acknowledgment of authors' contributions	D	✓
**Sample**		
Description	E	✓
Volume/mass of sample processed	D	✓
Microdissection or macrodissection	E	✓
Processing procedure	E	✓
If frozen, how and how quickly?	E	✓
If fixed, with what and how quickly?	E	N/A
Sample storage conditions and duration (especially for FFPE[b] samples)	E	✓
**Nucleic acid extraction**		
Procedure and/or instrumentation	E	✓
Name of kit and details of any modifications	E	✓
Source of additional reagents used	D	N/A
Details of DNase or RNase treatment	E	✓
Contamination assessment (DNA or RNA)	E	✓
Nucleic acid quantification	E	✓
Instrument and method	E	✓
Purity (A_{260}/A_{280})	D	✓
Yield	D	–
RNA integrity: method/instrument	E	✓
RIN/RQI or C_q of 3' and 5' transcripts	E	N/A
Electrophoresis traces	D	✓
Inhibition testing (C_q dilutions, spike, or other)	E	✓
**Reverse transcription**		
Complete reaction conditions	E	✓
Amount of RNA and reaction volume	E	✓
Priming oligonucleotide (if using GSP) and concentration	E	✓
Reverse transcriptase and concentration	E	✓
Temperature and time	E	✓
Manufacturer of reagents and catalogue numbers	D	✓
C_qs with and without reverse transcription	D[c]	✓
Storage conditions of cDNA	D	✓
**qPCR target information**		
Gene symbol	E	✓
Sequence accession number	E	✓
Location of amplicon	D	✓
Amplicon length	E	✓
In silico specificity screen (BLAST, and so on)	E	✓
Pseudogenes, retropseudogenes, or other homologs?	D	–

Sequence alignment	D	–
Secondary structure analysis of amplicon	D	✓
Location of each primer by exon or intron (if applicable)	E	✓
What splice variants are targeted?	E	✓
**qPCR oligonucleotides**		
Primer sequences	E	✓
RTPrimerDB identification number	D	N/A
Probe sequences	D[d]	N/A
Location and identity of any modifications	E	✓
Manufacturer of oligonucleotides	D	✓
Purification method	D	✓
**qPCR protocol**		
Complete reaction conditions	E	✓
Reaction volume and amount of cDNA/DNA	E	✓
Primer, (probe), Mg^{2+}, and dNTP concentrations	E	✓
Polymerase identity and concentration	E	✓
Buffer/kit identity and manufacturer	E	✓
Exact chemical composition of the buffer	D	N/A
Additives (SYBR Green I, DMSO, and so forth)	E	✓
Manufacturer of plates/tubes and catalog number	D	✓
Complete thermocycling parameters	E	✓
Reaction setup (manual/robotic)	D	✓
Manufacturer of qPCR instrument	E	✓
**qPCR validation**		
Evidence of optimization (from gradients)	D	✓
Specificity (gel, sequence, melt, or digest)	E	✓
For SYBR Green I, C_q of the NTC	E	✓
Calibration curves with slope and y intercept	E	✓
PCR efficiency calculated from slope	E	✓
CIs for PCR efficiency or SE	D	–
r^2 of calibration curve	E	✓
Linear dynamic range	E	✓
C_q variation at LOD	E	N/A
CIs throughout range	D	–
Evidence for LOD	E	N/A
If multiplex, efficiency and LOD of each assay	E	N/A
**Data analysis**		
qPCR analysis program (source, version)	E	✓
Method of C_q determination	E	✓
Outlier identification and disposition	E	✓
Results for NTCs	E	✓
Justification of number and choice of reference genes	E	✓
Description of normalization method	E	✓
Number and concordance of biological replicates	D	✓
Number and stage (reverse transcription or qPCR) of technical replicates	E	✓
Repeatability (intraassay variation)	E	✓

Reproducibility (interassay variation, CV)	D	–
Power analysis	D	–
Statistical methods for results significance	E	✓
Software (source, version)	E	✓
C_q or raw data submission with RDML	D	–

[a] All essential information (E) must be submitted with the manuscript. Desirable information (D) should be submitted if available. If primers are from RTPrimerDB, information on qPCR target, oligonucleotides, protocols, and validation is available from that source.
[b] FFPE, formalin-fixed, paraffin-embedded; RIN, RNA integrity number; RQI, RNA quality indicator; GSP, gene-specific priming; dNTP, deoxynucleoside triphosphate.
[c] Assessing the absence of DNA with a no–reverse transcription assay is essential when first extracting RNA. Once the sample has been validated as DNA free, inclusion of a no–reverse transcription control is desirable but no longer essential.
[d] Disclosure of the probe sequence is highly desirable and strongly encouraged; however, because not all vendors of commercial predesigned assays provide this information, it cannot be an essential requirement. Use of such assays is discouraged.

Supplementary Figures

Supplementary Fig. 1.

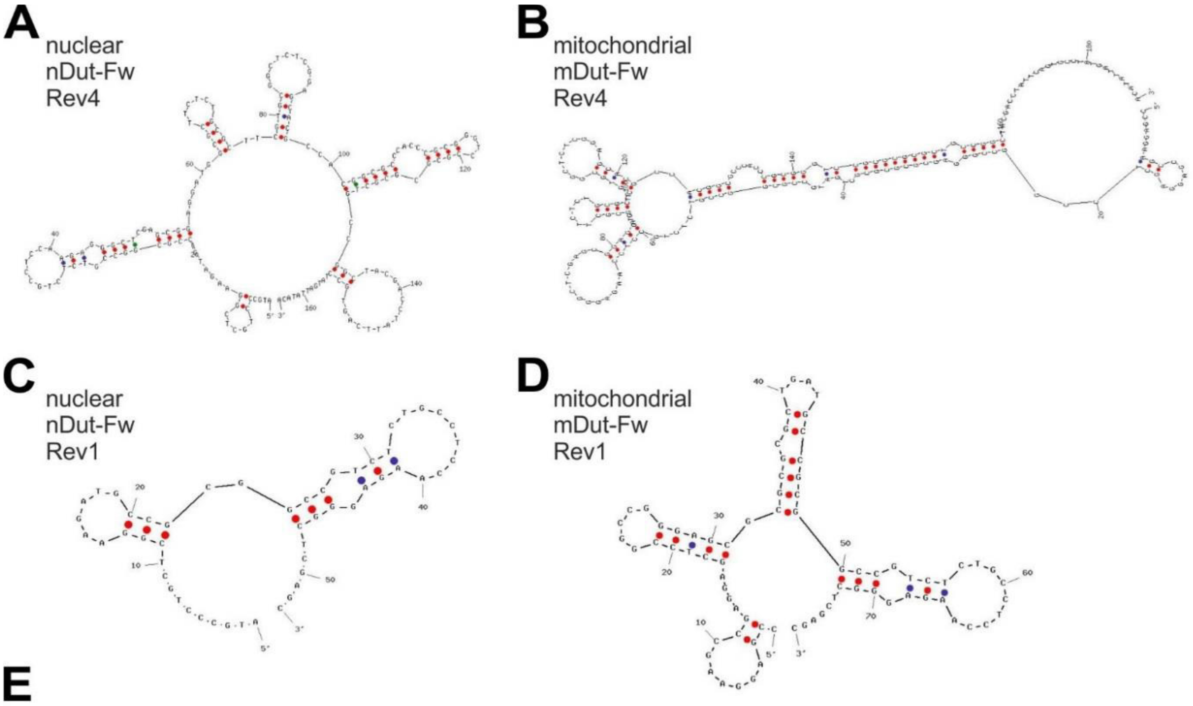

E

Isoform	Reverse	ΔG [kcal*mol^{-1}]	T_m [°C]	ΔH [kcal*mol^{-1}]	ΔS [kcal*K^{-1}*mol^{-1}]
nuclear	Rev4	-24.99	51.4	-307.1	-946.19
nuclear	Rev1	-5.37	46.6	-79.6	-248.96
mitochondrial	Rev4	-40.93	61.3	-377.5	-1128.86
mitochondrial	Rev1	-12.92	49.5	-170.2	-527.52

Supplementary Fig. 1. Possible secondary structures of the PCR products as predicted by the OligoAnalyzer tool of Integrated DNA Technologies. The introduced structures were selected based on the highest change in Gibbs free energy according to the tool. Blue circles indicate A-T interaction, red circles indicate G-C interaction, green indicates G-T interaction. **A B** Secondary structure of the nuclear (A) and mitochondrial (B) isoform with isoform-specific forward primers and Rev4 reverse primer. **C D** Secondary structure of the nuclear (C) and mitochondrial (D) isoform with isoform-specific forward primers and Rev1 reverse primer. **E** Table summarising the thermodynamic parameters of the four introduced structures.

Supplementary Fig. 2.

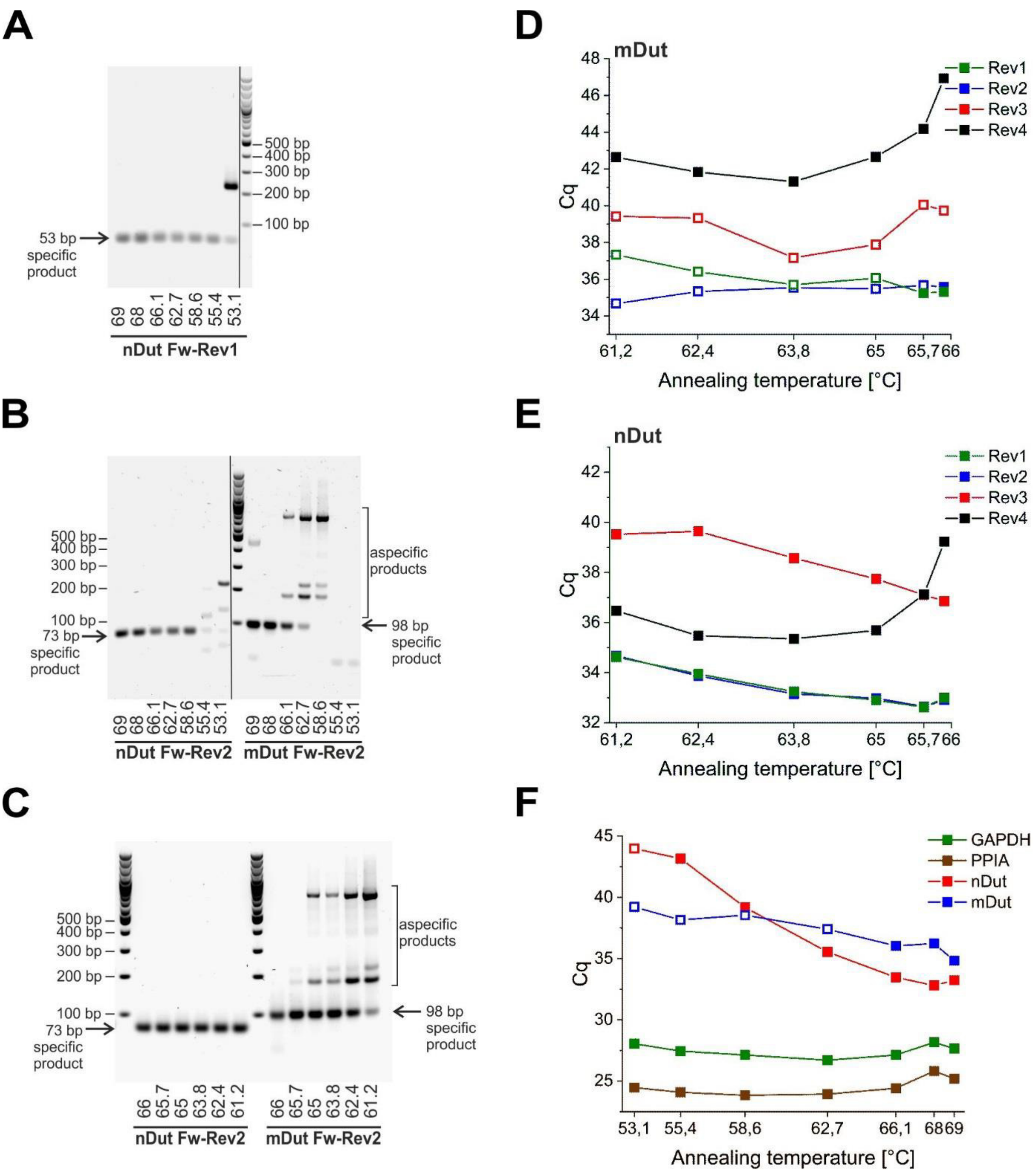

Supplementary Fig. 2. A Agarose gel electrophoresis of the PCR products for the nuclear isoform with Rev1 reverse primer at a range of annealing temperature from 53.1 to 69 °C . The specific product is indicated with an arrow. Vertical line indicates cropping of the image. **B C** Agarose gel electrophoresis of the PCR products for both nuclear and mitochondrial isoforms with Rev2 reverse primer at a range of annealing temperature from 53.1 to 69 °C (B) and from 61.2 to 66 °C (C). The specific products are indicated with arrows. Vertical line indicates cropping of the image. **D E** Quantification cycles (C_q) at a range of annealing temperatures from 61.2 to 66 °C with all four reverse primer candidates for the mitochondrial isoform (D) and nuclear isoform (E). The solid squares indicate specific products as determined with agarose gel electophoresis and melting curve analysis. Open squares indicate

aspecific products. **F** Quantification cycles (C_q) at a range of annealing temperatures from 53.1 to 69 °C with the selected primers. The solid squares indicate specific products as determined with agarose gel electophoresis and melting curve analysis. Open squares indicate aspecific products.

Supplementary Fig. 3.

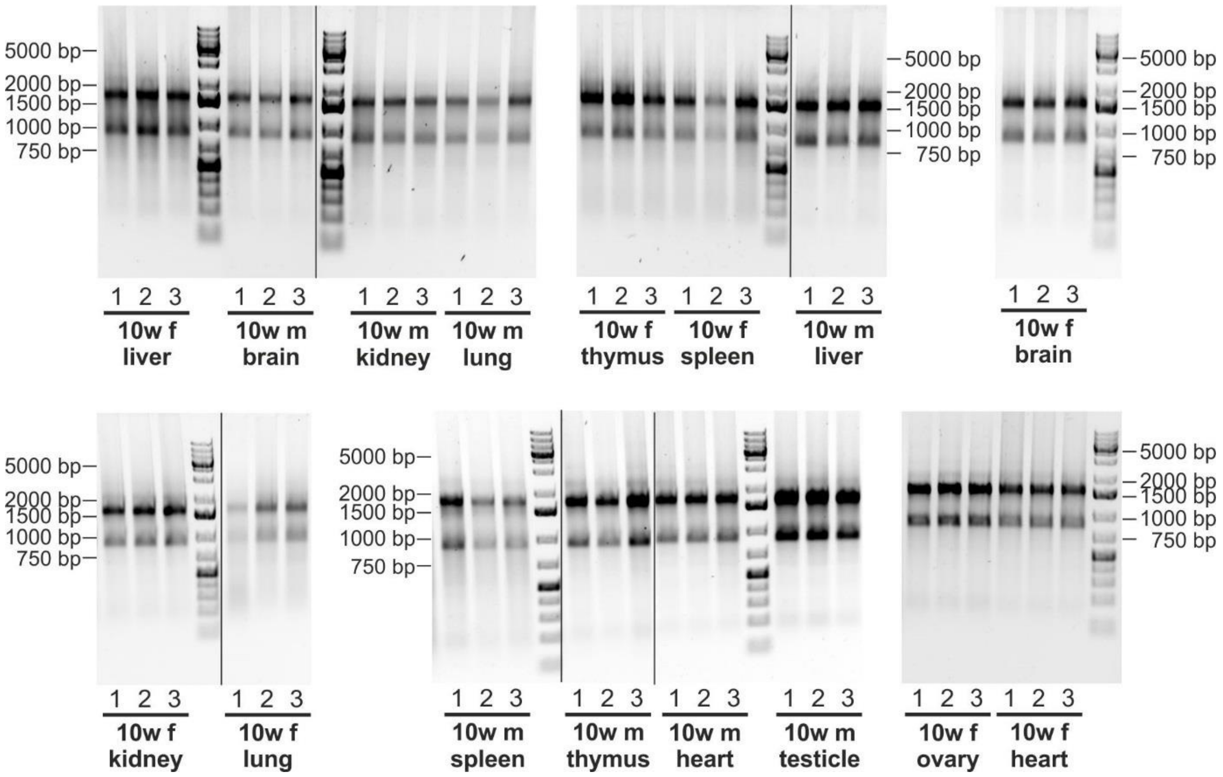

Supplementary Fig. 3. Agarose gel electrophoresis of the RNA samples used in this study. Vertical lines indicate cropping of the image.

Supplementary Fig. 4.

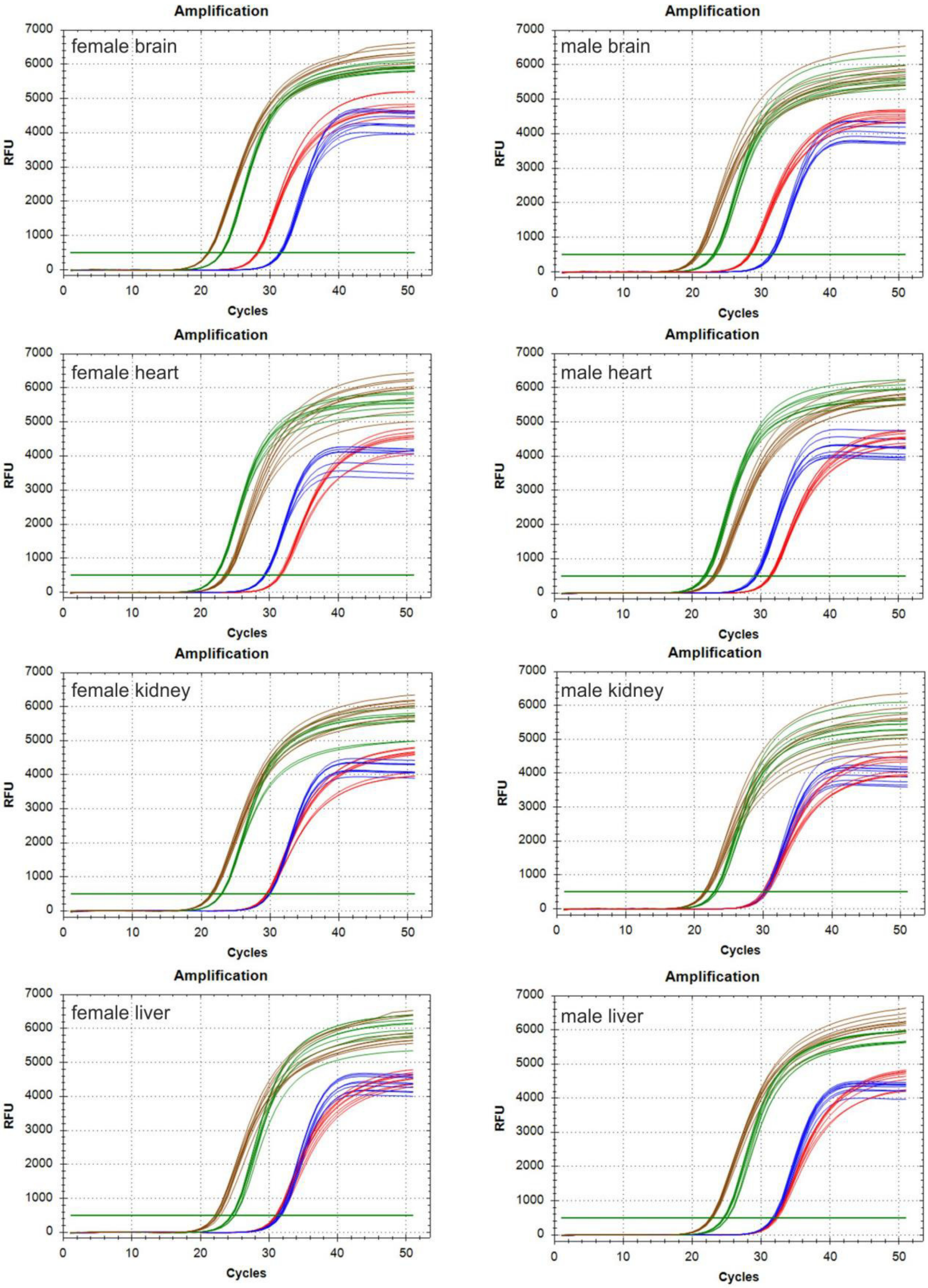

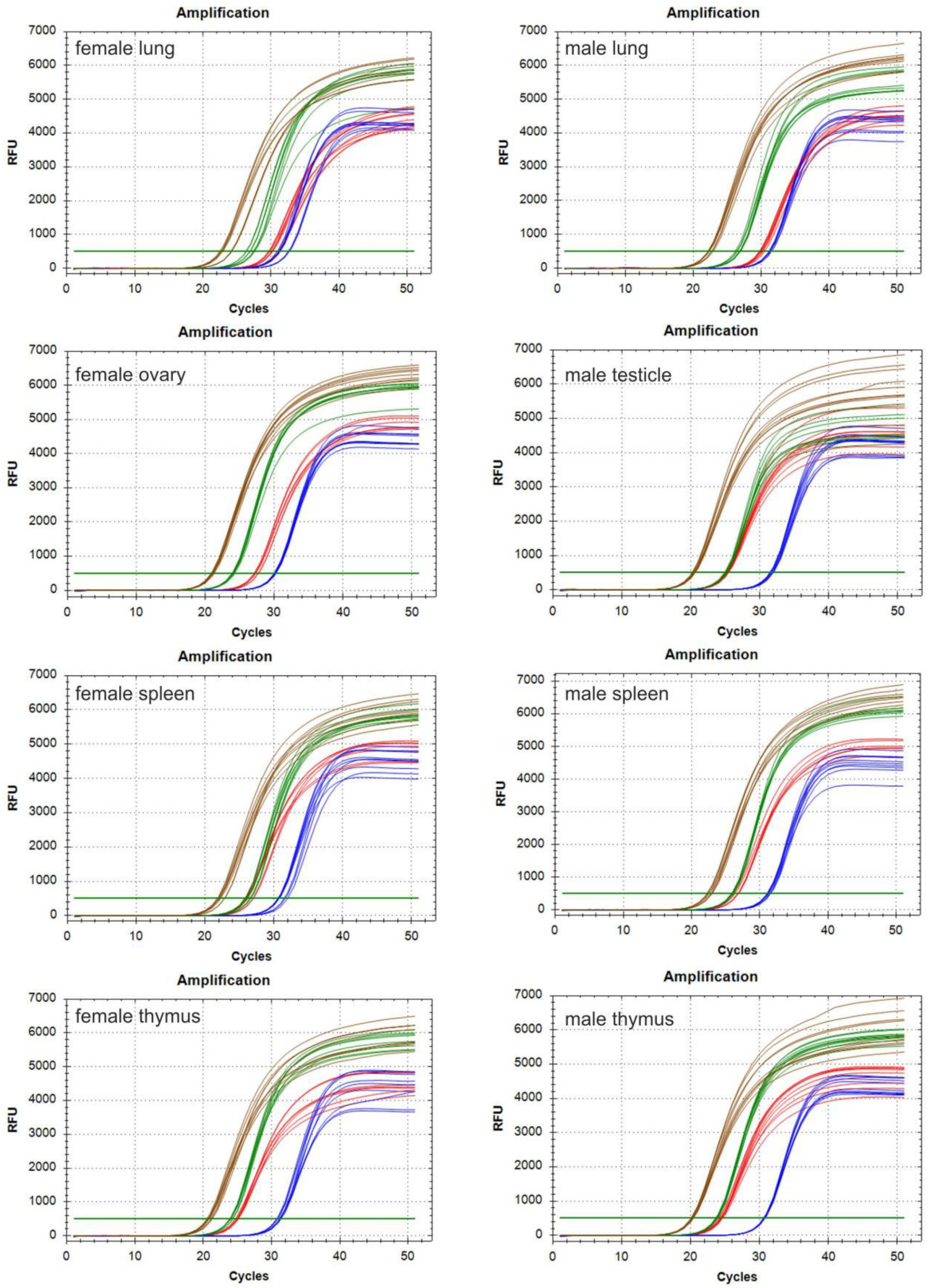

Supplementary Fig. 4. Amplification curves of all the biological groups. Red colour indicates the nuclear isoform, blue indicates the mitochondrial isoform, green indicates GAPDH and brown indicates PPIA. Three biological replicates and three technical replicates for each biological replicates are shown for each graph. Threshold values are set to 500 RFU.

RFU, relative fluorescent unit. The biological groups of the same organs are juxtaposed with the female sex first.
